# Approaches in Managing Resistant Hypertension: A Review

**DOI:** 10.7759/cureus.57804

**Published:** 2024-04-08

**Authors:** Sanjay Mittal, Peeyush Jain, Rajat Sharma, Chandrashekhar K Ponde, Satyanarayan Routray, Sandeep Chopra, Rohit Kumar, Syed Naqvi, Rajan Mittal

**Affiliations:** 1 Clinical and Preventive Cardiology Department, Medanta Hospital, Gurugram, IND; 2 Noninvasive Cardiology Department, Fortis Okhla, Delhi, IND; 3 Heart Rhythm and Pacemaker Division, Fortis Hospital, Mohali, IND; 4 Cardiology Department, Apollo Hospital, Bhubaneswar, IND; 5 Internal Medicine Department, Srirama Chandra Bhanja (SCB) Medical College and Hospital, Cuttack, IND; 6 Internal Medicine Department, Fortis Hospital, Ludhiana, IND; 7 Medical Affairs Department, Dr. Reddy's Laboratories, Hyderabad, IND

**Keywords:** ace inhibitors and angiotensin receptor blockers, thiazide diuretics, calcium channel blockers, antihypertensive agents, blood pressure, treatment-resistant hypertension

## Abstract

In India, around 234 million adults (one in three) suffer from hypertension (HTN). An average of 10% of these cases are likely to be resistant hypertension (RH). This load of 23 million patients is expected to expand further with revisions in diagnostic criteria. The treatment and control rates of hypertension in India average around 30% and 15%, respectively.

Pharmacological management involves a stepwise approach starting with optimizing the A-C-D (angiotensin-converting enzyme inhibitors (ACEIs) or angiotensin receptor blockers (ARBs), calcium channel blockers (CCBs), and thiazide-like diuretics) triple-drug combination, followed by substitution with a thiazide-like diuretic and use of spironolactone as a next step (fourth drug). The subsequent steps are suggestions based on expert input and must be individualized. These include using a β-blocker as the fifth drug and an α1-blocker or a peripheral vasodilator as a final option when target blood pressure (BP) values are not achieved. Sodium-glucose cotransporter-2 inhibitors (SGLT2i) are likely to be helpful in managing RH due to their renal and cardiovascular protection as well as mortality benefits. SGLT2i lowers BP independent of the dosage and concomitant anti-hypertensive medications. Patient education and tools to monitor BP and treatment compliance will improve outcomes with these medications.

In addition to therapeutic intervention, a preventive approach for RH mandates a need to identify patients at risk and use appropriate preventive and optimal therapy to prevent uncontrolled hypertension in patients with cardiovascular disorders.

## Introduction and background

Hypertension (HTN), often called the "silent killer," is a leading preventable cause of cardiovascular disease (CVD) and associated morbidity and mortality. Worldwide, 1.2 billion patients are impacted by hypertension. This number has doubled in the past 30 years with a significant contribution from low- and middle-income countries (one billion, 82% of global HTN patients) [1]. The overall prevalence of HTN in India stands at 29.8%, with significant variations observed between rural (27.6%) and urban (33.8%) areas. Alarmingly, about 12% of individuals with HTN in India manage to keep their blood pressure (BP) under control. Uncontrolled HTN poses a significant risk factor for cardiovascular diseases such as heart attacks and strokes, contributing to one-third of total deaths in India [2].

Definition of RH

**Figure 1:**
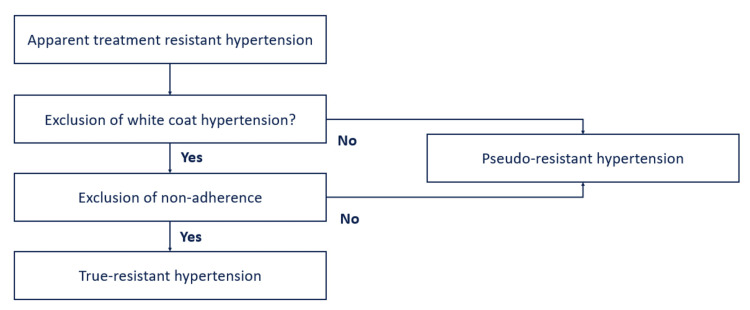
Definition of resistant hypertension

Despite concerted efforts of physicians, the healthcare system, and patients, globally, only 14% of the 1.2 billion patients achieve and maintain target blood pressure. The American Heart Association/American College of Cardiology (AHA/ACC) and the International Society of Hypertension (ISH) define resistant hypertension (RH) as failure to achieve blood pressure (BP) of <130/80 mmHg (average of two readings at a healthcare clinic on two different or consecutive days) or average BP of <125/75 mmHg on a 24-hour ambulatory BP monitor (ABPM), with use of triple-drug therapy. The three drugs should be of different classes (preferably Ca++ channel blocker + angiotensin-converting enzyme inhibitors (ACEIs)/angiotensin receptor blocker (ARB) + thiazide diuretic (often referred to as A-C-D combination)) at maximally recommended or tolerated dose and frequency. Hypertensive patients are labeled as controlled RH when they achieve a BP of <130/80 mmHg with >4 drugs [1]. Thus, based on AHA/ACC criteria, RH patients can be either uncontrolled or controlled based on the number of anti-hypertensive medications (Figure 1) [3].

Types and BP monitoring

Resistant HTN can be either true/pseudo/apparent or secondary. True resistant HTN is defined as a properly measured office BP > 140/90 mmHg with a mean 24-hour ambulatory BP > 130/80 mmHg in a patient confirmed to be taking ≥3 anti-hypertensive medications. The term apparent treatment RH (aTRH) is used when ≥1 of the following data elements are missing: medication dose, adherence, or out-of-office BP.

Pseudo-treatment RH occurs either due to poor compliance with prescribed medications, white coat effect (high BP in the clinic (higher by 20/10 mmHg) and near-normal or normal BP readings at home or outside the healthcare setting), or suboptimal treatment or incorrect technique to measure BP (e.g., wrong cuff size or non-calibrated digital device) (Figure 1) [4].

**Figure 2:**
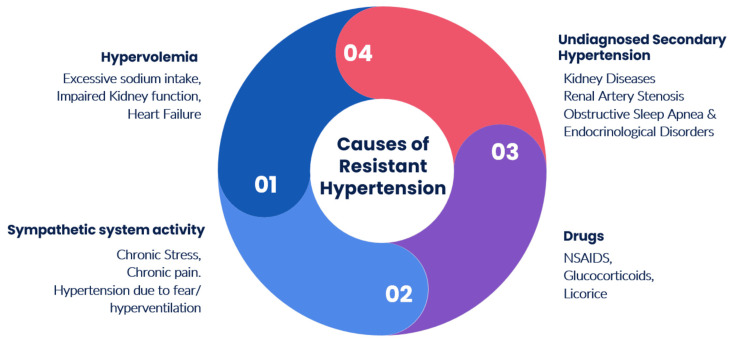
Causes of resistant hypertension NSAIDs: non-steroidal anti-inflammatory drugs

Secondary causes of RH occur due to an underlying endocrine, renal artery stenosis, and primary aldosteronism, and obstructive sleep apnea. Several drugs can induce or exacerbate pre-existing HTN, with non-steroidal anti-inflammatory drugs (NSAIDs) being the most common due to their wide use (Figure 2).

The prevalence and incidence of resistant HTN often get confounded (on the higher side) due to the presence of pseudo-resistant hypertension. Pseudo-resistant HTN, often mistaken for treatment-resistant cases, arises from factors such as medication non-compliance and inadequate drug therapy rather than inherent resistance to treatment. Contributing factors such as lifestyle adherence, measurement techniques, white coat effect, and medications impacting blood pressure regulation also play significant roles in apparent treatment resistance (Figure 2). Bhatt et al. [5] and de la Sierra et al. [6] have shown that 33% and 37% of RH patients in their respective study populations have pseudo-RH. False high BP readings occur as compliance with correct BP measurement techniques has been observed in only around 70% of clinical settings [7]. Hence, enhancing awareness and implementing accurate BP recording methods will prevent pseudo-resistant HTN.

Chaos theory: Pseudo-treatment RH versus true treatment RH

Blood pressure in an individual is influenced by several external (environmental climate, temperature, and time of the day) and internal (diurnal, postural, and emotional) factors. It has led to the origin of chaos theory, as these factors and the resultant changes in BP are complex and unpredictable [8]. These chaotic shifts in BP lead to an erroneous diagnosis of resistant HTN (RH) in patients on anti-hypertensive medications. Hence, one needs to be aware of the possibility of chaos in BP before labeling a patient as true treatment RH.

Epidemiology

Globally, RH is observed in 3%-30% of hypertensive patients [3]. Published data from India shows that RH is found in 11%-16.13% of hypertensive patients [9,10]. With 234 million hypertensive patients (one in three adults in India), around 23 million are likely to have RH due to non-adherence to treatment and therapeutic inertia [11].

The prevalence of HTN varies from 24% to 59% across India. Certain parts, such as the eastern region, especially Assam, have a higher figure due to excess salt, alcohol, and khaini (smokeless tobacco) consumption among tea plantation workers. Low awareness of diagnosis, low treatment, and poor control rates impact the treatment landscape in India [2].

RH is more likely to occur as age advances and in patients with chronic kidney disease (CKD), obesity, and obstructive sleep apnea. As these risk factors are on the rise globally, they are likely to increase RH prevalence [12]. The other factors predisposing a patient to develop RH are gender (female sex), excess sodium intake, and use of NSAIDs [13,14].

## Review

Pathophysiology and complications of RH

The three significant physiological changes in RH include increased sodium and fluid overload, overactivity of the renin-angiotensin-aldosterone system, and overactivity of the sympathetic nervous system [15]. The additional role of arterial stiffness and gut dysbiosis has been shown recently. Diuretics and aldosterone antagonists are used as a triple-drug regimen and the fourth drug in treating RH. Other medication approaches are governed by the underlying mechanism of TRH and the patient's clinical profile.

These RH patients are at a higher risk of developing cardiovascular and renal complications, i.e., myocardial infarction, heart failure (HF), stroke, death, and CKD, compared to non-RH patients. This risk is influenced by comorbidities and is 47%-100% higher than in non-RH patients. Gupta et al. [16] have shown that 57% and 24% of stroke and coronary diseases occur, respectively, due to uncontrolled BP in India. Hence, early diagnosis and treatment of RH are likely to reduce cardiovascular and renal complications and associated mortality [17]. Recent studies have shown that RH leads to a significant decline in quality of life, primarily physical functioning [17]. Patient engagement and education, along with treatment approaches, will help address these concerns. Health-related quality of life should be an integral component of managing RH.

AHA issued the first guideline on RH in 2008. Since then, multiple studies have shown that outcomes of patients with RH are poor compared to non-RH patients. RH patients are 47% more likely to develop the cumulative outcome of death, myocardial infarction, heart failure, stroke, or CKD over 3.8 years. The risk of end-stage renal disease, ischemic heart event, heart failure, stroke, and death is increased by 32%, 24%, 46%, 14%, and 6%, respectively, in RH patients compared to non-RH subjects. Studies using ABPM have shown a twofold increased risk of CVD events in RH patients. In RH patients, comorbid conditions tend to impact the outcomes negatively. Patients with CKD and RH are at a higher risk of myocardial infarction, stroke, peripheral arterial disease, heart failure, and all-cause mortality. Ischemic heart disease patients with RH have a higher probability of death, myocardial infarction, and stroke.

RH patients who achieve control of their BP have a lower risk for cardiovascular events. The Reasons for Geographic and Racial Differences in Stroke (REGARDS) study has shown a twofold increased risk of coronary heart disease in uncontrolled RH patients compared with controlled RH patients [18].

Diagnosis of RH

The European Society of Cardiology (ESC)/European Society of Hypertension (ESH) and the National Institute for Health and Care Excellence (NICE) have maintained the earlier recommendation of 140/90 mmHg as a cutoff for HTN (Table 1) [19,20]. RH, defined as BP of >140/90 mmHg in a patient on three or more anti-hypertensive drugs (one being a diuretic), has also not been altered. The prevalence of RH using these criteria is ≈10%. In contrast, Canadian guidelines have recommended BP levels above the target based on comorbidities and projected cardiovascular risk for defining RH patients [21]. These patients should have been on three or more anti-hypertensive drugs at optimal doses, with one of those agents preferably being a diuretic [21].

**Table 1:** Comparison of existing guidelines for the diagnosis of resistant hypertension BP: blood pressure, SBP: systolic blood pressure, DBP: diastolic blood pressure, ESH/ESC: European Society of Hypertension/European Society of Cardiology, AHA/ACC: American Heart Association/American College of Cardiology, ACEI: angiotensin-converting enzyme inhibitor, ARB: angiotensin receptor blocker, CCB: calcium channel blocker

Guidelines	ESH/ESC (2023)	AHA/ACC (2018)	Hypertension Canada (2020)
BP criteria			
SBP	>140 mmHg	>130 mmHg	Above target
DBP	>90 mmHg	>80 mmHg	Above target
Number of anti-hypertensive medications	>3	>3	>3
Anti-hypertensive class	Ca^++^ channel blocker + ACEI/ARB + thiazide diuretic (often referred to as A-C-D combination)	A-C-D combination	Three or more different classes (including a diuretic)

Untreated obstructive sleep apnea is strongly linked to HTN, particularly prevalent in patients with resistant HTN. In a cross-sectional study of 41 patients with treatment-resistant HTN, 83% were diagnosed with sleep apnea based on an apnea-hypopnea index ≥ 10 events/hour. The result suggested that the severity of sleep apnea is inversely related to blood pressure control, despite the use of multiple medications (Table 1) [22].

Importance of Early Diagnosis

A detailed history and physical examination are often missed in a busy outpatient setting for RH. It delays identifying the clinical features and subsequent diagnostic workup to identify secondary causes of RH. Hence, BP should be measured in both arms (Table 2), and auscultation should be done for the carotid, renal, and heart sounds and murmurs [23]. It will aid in the early identification of secondary causes of RH, especially in young adults, and reduce the CVD risk (Table 2).

**Table 2:** Recommendations for BP measurement HTN: hypertension, BP: blood pressure

Patient	Empty bladder
	The patient needs to rest in a seated position for five minutes
	Do not talk or text
Position	Sit with back supported
	Keep both feet flat on the floor
	Uncrossed legs
Patient training	Provide information about HTN diagnosis and management
	Provide information on the proper selection of a device
	Provide instruction if other BP readings should be brought to healthcare visits
Setting	Quiet place
	Appropriate temperature
	Supported back, arms, and feet of a patient
Device	Recording from both upper arms
	Cuff should be pulled taut, with comparable tightness at the top and bottom edges of the cuff, around the bare upper arm
	Cuff wrapped directly on the skin at the heart level

Challenges in India

Only 48% and 43% of urban and rural community patients (18-49 years) are aware of their disease condition despite underlying HTN [13]. The awareness and treatment rates have increased from a low of 13% and 9% in 1995 to 56% and 36% by 2015, respectively [16]. Despite this positive change, control rates have moved from 2% (1995) to only 21% (2015). Factors such as access and affordability to healthcare settings and treatment options and increased dietary sodium play a role in the development of RH [24].

Kothavale et al. [24], in a study of 28,019 elderly, determined the dropouts from HTN care in India: compliance across the spectrum of measurements of BP (72.5%), awareness/diagnosis of their HTN (57.3%), receiving treatment (50.5%), and control with intervention measures (27.5%). The highest dropout from medical care occurred at the control stage of disease management. Probable factors contributing to these dropouts were socioeconomic, demographic, and lifestyle factors.

Factors such as caste, religion, living situation, monthly per capita expenditure (MPCE) quintile, location, family history of hypertension, employment status, physical inactivity, and alcohol consumption emerged as significant predictors of uncontrolled hypertension. Additionally, research by Mahapatra et al. [9] indicates that prolonged hypertension duration, obesity, disordered sleep history, and elevated fasting blood glucose levels correlate with an increased risk of resistant hypertension (RH). Moreover, there is a notable prevalence of therapeutic inertia among healthcare providers, highlighting the need for ongoing education and training to address this issue.

Management of RH

Patients with RH have heterogeneous pathophysiological perspectives, and non-pharmacological interventions should be an integral part of the management. These include measures such as reduced consumption of Na+ (<1,500 mg/day) (mainly reduce processed food), dietary potassium supplementation (3,500-5,000 mg/day as a dietary constituent), weight loss, regular exercise (mix of aerobic, dynamic, and isometric), healthy sleep hygiene, and stress management, which are effective in the management of RH [4].

Pharmacological Management of RH

Angiotensin-converting enzyme inhibitors (ACEIs) or angiotensin receptor blockers (ARBs), calcium channel blockers (CCBs), and thiazide-like diuretics are first-line drugs for the treatment of RH. It is commonly referred to as the A-C-D combination. The definition of RH requires three drugs at optimal doses, including a diuretic, and would usually mean including anti-hypertensives from the A-C-D combination (Table 3).

**Table 3:** Once daily, first-line anti-hypertensive agents by pharmacological class used to optimize the three-drug regimen in patients with resistant hypertension ACEI: angiotensin-converting enzyme inhibitor, ARB: angiotensin receptor blocker, CCB: calcium channel blocker

Drug class	Regimens
Thiazide diuretics, thiazide-like diuretic	Hydrochlorothiazide 12.5-50 mg/day, chlorthalidone 12.5-25 mg/day, indapamide 1.25-2.5 mg/day
ACEI	Benazepril 10-40 mg/day, enalapril 5-40 mg/day, fosinopril 10-40 mg/day, lisinopril 10-40 mg/day, moexipril 7.5-30 mg/day, perindopril 4-16 mg/day, quinapril 10-80 mg/day, ramipril 2.5-20 mg/day
ARB	Azilsartan 40-80 mg/day, candesartan 8-32 mg/day, eprosartan 600-800 mg/day, irbesartan 150-300 mg/day, losartan 50-100 mg/day, olmesartan 20-40 mg/day, telmisartan 20-80 mg/day, valsartan 80-320 mg/day
CCB (dihydropyridine)	Amlodipine 2.5-10 mg/day, felodipine 2.5-10 mg/day, nifedipine LA 30-90 mg/day, nisoldipine 17-34 mg/day
CCB (non-dihydropyridine)	Diltiazem 120-360 mg day, verapamil SR 120-360 mg/day

AHA/ACC guidelines recommend strict goal-based control of BP as the benefits outweigh the safety concerns. It aligns with the Systolic Blood Pressure Intervention Trial (SPRINT) and Action to Control Cardiovascular Risk in Diabetes (ACCORD) subgroup analysis [25]. European guidelines, in contrast, differ as they try to balance safety with the benefits of treatment intensification [19]. Pharmacological treatment aims for effective yet simple drug regimens that patients can easily follow, typically involving once-daily doses of drug combinations. In uncontrolled RH patients, spironolactone, a mineralocorticoid (MRA) receptor blocker drug, is preferred as the fourth-line agent (Table 3). The Prevention and Treatment of Hypertension with Algorithm Based Therapy-2 (PATHWAY-2) study has shown that the efficacy of spironolactone is superior to the efficacy of β-blocker and doxazosin (α-blocker) [26]. It is administered once daily and can be initiated with a small dose of either 12.5 or 25 mg.

In addition, sodium-glucose cotransporter-2 inhibitors (SGLT2i) are likely to be helpful in managing RH due to their renal and cardiovascular protection as well as mortality benefits [21]. These glucose-lowering drugs lower BP independent of the dosage and concomitant anti-hypertensive medications. They improve outcomes in patients (including non-diabetics) with proteinuria, CKD, or HF [27,28].

A post hoc analysis of the EMPA-REG outcome study showed that empagliflozin was superior to placebo in RH patients (38% (empagliflozin) versus 26% (placebo) patients achieved systolic BP < 130 mmHg at week 12 of follow-up) [28]. A significant reduction in CV death, or hospitalization for HF, and incident or progressive nephropathy were observed with empagliflozin. A similar analysis of the Canagliflozin and Renal Events in Diabetes with Established Nephropathy Clinical Evaluation (CREDENCE) study showed benefits and better long-term outcomes with canagliflozin for RH patients [29]. There is thus a role for flozins in RH patients with comorbid diabetes mellitus (DM), CKD, albuminuria, and HF (Table 4).

**Table 4:** Clinical data of spironolactone in RH BP: blood pressure, SBP: systolic blood pressure, DBP: diastolic blood pressure, CKD: chronic kidney disease, GFR: glomerular filtration rate, RH: resistant hypertension, PATHWAY-2: Prevention and Treatment of Hypertension with Algorithm Based Therapy-2, TOPCAT: Treatment of Preserved Cardiac Function Heart Failure With an Aldosterone Antagonist, HFpEF: heart failure with preserved ejection fraction, ReHOT: Resistant Hypertension Optimal Treatment, ASPIRANT: Addition of Spironolactone in Patients With Resistant Arterial Hypertension, ABPM: ambulatory BP monitor ^*^Spironolactone versus placebo, bisoprolol, and doxazosin to determine the optimal treatment for drug-resistant hypertension ^$^TOPCAT [30] ^@^ReHOT [31]

Reference	Study name, year	Number	Resistant hypertension inclusion criteria	Intervention	Treatment duration	Results (efficacy and safety)
Williams et al. [26]	Spironolactone versus placebo, bisoprolol, and doxazosin to determine the optimal treatment for drug-resistant hypertension (PATHWAY-2): a randomised, double-blind, crossover trial, 2015	335	Clinic SBP > 140 mmHg, home SBP > 130 mmHg, three drugs for >3 months	Spironolactone (25-50 mg), bisoprolol (5-10 mg), doxazosin modified release (4-8 mg), and placebo, as an add-on treatment	12 weeks	Spironolactone > placebo/doxazosin/bisoprolol (home SBP) (p < 0·0001), all treatments were well tolerated
Tsujimoto et al. [30]	Spironolactone use and improved outcomes in patients with heart failure with preserved ejection fraction with resistant hypertension^$^, 2020	3,441	SBP ≥ 130 mmHg and/or DBP ≥ 80 mmHg, A-C-D triple-drug therapy, or ≥4 drugs from different classes	Spironolactone versus placebo: patients of HFpEF with (n = 1,004) and without (n = 2,437) RH; the primary outcome was a composite of cardiovascular death, aborted cardiac arrest, or heart failure hospitalization	3.3 years	Spironolactone > placebo (p = 0.009) (patients with HFpEF with RH), spironolactone = placebo (p = 0.97) (patients with HFpEF without RH)
Krieger et al. [31]	ReHOT spironolactone versus clonidine as a fourth-drug therapy for resistant hypertension: the ReHOT Randomized Study (Resistant Hypertension Optimal Treatment), 2018	1,597	Uncontrolled BP, three drugs for >3 months	Spironolactone (12.5-50 mg QD) or clonidine (0.1-0.3 mg BID)	12 weeks	Spironolactone = clonidine (p = 1.00), spironolactone > clonidine (more significant decrease in 24-hour systolic and diastolic BP and diastolic daytime ambulatory BP)
Rossignol et al. [32]	Spironolactone and resistant hypertension in heart failure with preserved ejection fraction, 2018	403	SBP: 140-160 mmHg, >3 drugs	Spironolactone versus placebo	8 months	Spironolactone > placebo (SBP and DBP) (p = 0.003)
Oxlund et al. [33]	Low dose spironolactone reduces blood pressure in patients with resistant hypertension and type 2 diabetes mellitus: a double blind randomized clinical trial, 2013	119	Uncontrolled SBP/DBP (>130/80 mmHg), three-drug therapy	Spironolactone (25 mg/50 mg) or placebo	16 weeks	Spironolactone > placebo (significant BP and urinary albumin/creatinine ratio lowering effects), hyperkalemia (dose reduction (n = 3) and discontinuation (n = 1))
Vaclavik et al. [34]	Effect of spironolactone in patients with resistant arterial hypertension in relation to age and sex: insights from the ASPIRANT trial, 2014	111	Uncontrolled SBP/DBP (>140/90 mmHg), three-drug therapy (including a diuretic)	Spironolactone versus placebo	8 weeks	Spironolactone > placebo (patients with a median age > 62 years), spironolactone = placebo (patients aged ≤62 years)
Václavík et al. [35]	Effect of spironolactone in resistant arterial hypertension: a randomized, double-blind, placebo-controlled trial (ASPIRANT-EXT), 2014	161	Uncontrolled SBP/DBP (>140/90 mmHg), three-drug therapy (including a diuretic)	Spironolactone versus placebo	8 weeks	Spironolactone > placebo (significant decrease of both SBP and DBP and markedly improves BP control)
de Souza et al. [36]	Efficacy of spironolactone therapy in patients with true resistant hypertension, 2010	175	True resistant hypertension using ABPM	Spironolactone (25-100 mg/ day)	15 months	Effective and safe in decreasing blood pressure, especially in those with abdominal obesity and lower arterial stiffness
Chapman et al. [37]	Effect of spironolactone on blood pressure in subjects with resistant hypertension, 2007	1,411	Uncontrolled BP on three drugs	Spironolactone	1.3 years	Spironolactone effectively lowers blood pressure in RH patients, well tolerated (6% of participants discontinued due to adverse events)
Václavík et al. [38]	Addition of spironolactone in patients with resistant arterial hypertension (ASPIRANT): a randomized, double-blind, placebo-controlled trial, 2011	117	Uncontrolled SBP/DBP (>140/90 mmHg), three-drug therapy (including a diuretic)	Spironolactone versus placebo	8 weeks	Spironolactone > placebo (SBP), well tolerated
Abolghasmi et al. [39]	Efficacy of low dose spironolactone in chronic kidney disease with resistant hypertension, 2011	41	Uncontrolled BP with A-C-D combination in patients with moderately severe CKD (GFR: 25-50 mL/minute)	Spironolactone 25-50 mg/day versus placebo	12 weeks	Spironolactone > placebo (significant additive BP reduction in CKD patients (stage 2 and 3) with RH)

Guideline Recommendations

American and European guidelines for the management of RH have similar recommendations for both non-pharmacological (patient-appropriate lifestyle changes) and pharmacological (stepwise recommendation for drug combinations (in terms of classes of agents and their respective orders)) [19]. There are only minor differences, such as AHA/ACC guidelines emphasizing a minimum of six hours of sleep, as there is emerging data on the correlation between shorter sleep and the occurrence of HTN/resistant HTN. ESC/ESH guidelines currently do not have sleep-related lifestyle recommendations. Pharmacological management as per AHA/ACC and ESC/ESH guidelines involves a stepwise approach starting with optimizing the A-C-D triple-drug combination, followed by substitution with a thiazide-like diuretic and use of spironolactone as a next step (fourth drug) [37]. The subsequent steps are suggestions based on expert input and must be individualized. These include using a β-blocker as the fifth drug and an α1-blocker or a peripheral vasodilator as a final option when target blood pressure values are not achieved.

The effectiveness of combining α‐/β‐blockade for treating neurogenic HTN and RH patients is evident, as it blocks sympathetically mediated increases in both cardiac output and peripheral resistance. However, this approach may be hindered partly due to negative perceptions of α‐blockers. Intravenous labetalol, which bypasses first‐pass hepatic metabolism, demonstrates clear efficacy in managing neurogenic hypertension during clonidine withdrawal and is a cornerstone in hypertension treatment post-acute stroke. Additionally, α‐blockers have shown effectiveness as supplementary therapy in resistant hypertension [40]. In a retrospective study of 97 patients with RH, doxazosin was added as the fifth anti-hypertensive medication for those not responding well to or tolerating other drug combinations. Doxazosin dosage ranged from 2 to 16 mg/day, with an average follow-up of 21+/-17 months. Analysis of data from 34 patients showed a significant reduction in blood pressure from 159+/-20/92+/-14 to 126+/-16/73+/-10 mmHg, indicating that doxazosin is both well tolerated and effective for patients with resistant arterial HTN requiring multiple anti-hypertensive medications [41].

ESH 2023 guideline recommends a three-drug combination comprising a CCB, thiazide/thiazide-like diuretics, and either an ACEI or an ARB. The guideline also recommends managing resistant HTN as a high-risk condition, because it is frequently associated with HTN-mediated organ damage (HMOD) and increased CVD risk [20].

Stepwise Approach

**Figure 3:**
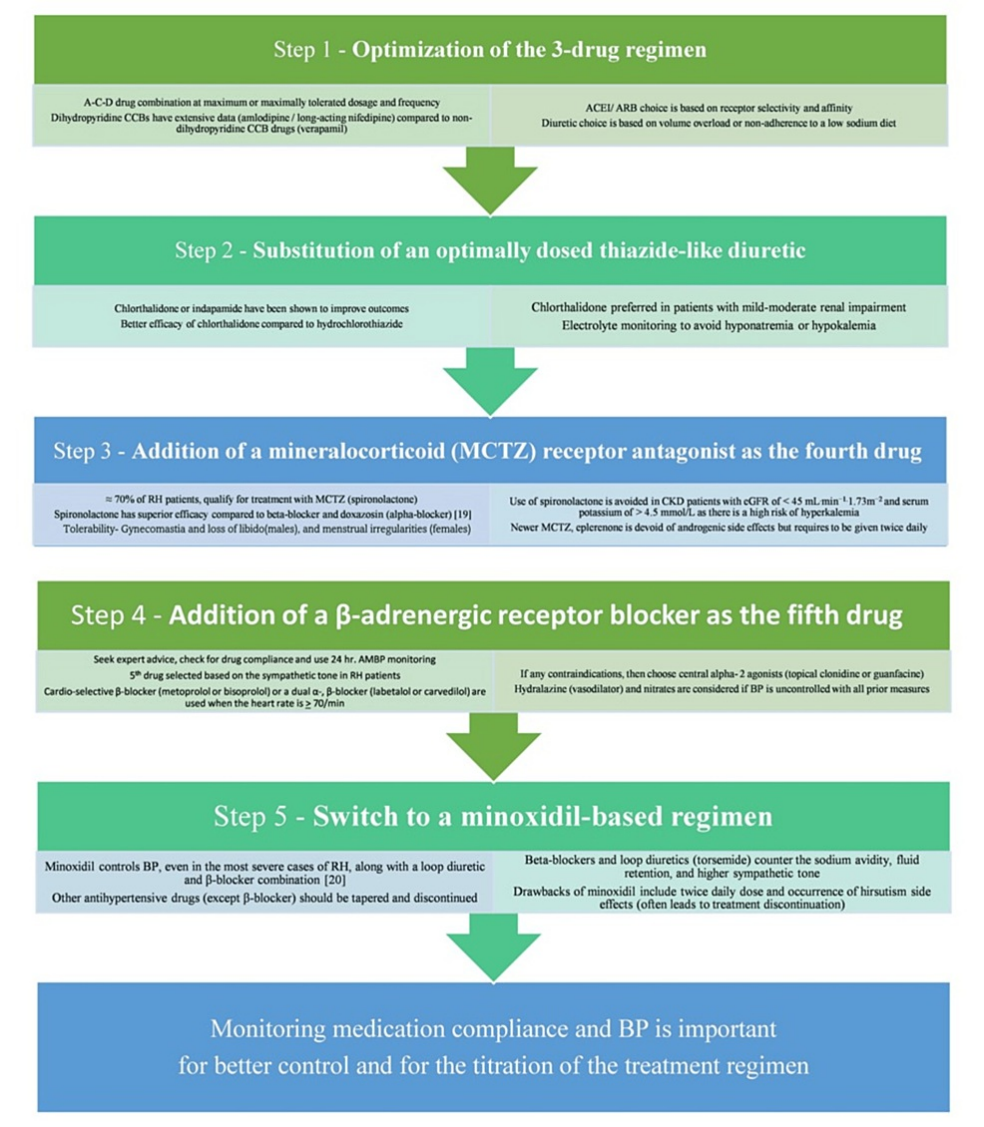
Five-step process for managing RH BP: blood pressure, RH: resistant hypertension, CCB: calcium channel blocker, ACE: angiotensin-converting enzyme, ARB: angiotensin receptor blocker, MCTZ: mineralocorticoid, CKD: chronic kidney disease, eGFR: estimated glomerular filtration rate, ABPM: ambulatory BP monitor

The desired goal in managing RH can be achieved by using therapeutic interventions in a phase-wise manner (five-step process) (Figure 3) [4].

CVD and RH

One-third of patients with cardiovascular conditions (e.g., coronary artery disease (CAD)) are likely to have or develop RH [42]. These patients have more severe vascular disease resulting in a higher risk of major cardiovascular events and all-cause mortality than controlled HTN. There is thus a need to identify patients at risk of RH and use appropriate preventive and optimal therapy to prevent uncontrolled HTN in patients with cardiovascular disorders.

CKD and RH

CKD and RH have a bidirectional relationship, resulting in a greater risk of end-organ damage [43]. Thus, RH is more likely to occur in CKD patients with higher risk associated with declines in glomerular filtration rate (GFR) and sodium excretion, and RH alone can also result in CKD. This situation is further compounded due to multiple comorbidities in CKD patients, resulting in a higher pill burden. Some common co-medications, such as non-steroidal anti-inflammatory drugs and aspirin, are likely to blunt the actions of anti-hypertensive medications.

The first step in managing RH in CKD patients is to confirm the presence of true treatment-resistant HTN and rule out pseudo-resistance. The following steps involve identifying reversible or interfering factors, excluding secondary causes and end-organ damage. Lifestyle and pharmacological measures should be initiated in collaboration with a nephrologist.

RH and Heart Failure With Reduced Ejection Fraction

RH patients are at higher risk of developing heart failure with reduced ejection fraction (HFrEF). The burden of comorbid RH and HFrEF is also increasing globally with the number of RH patients. Management of these patients includes lifestyle measures such as individualized exercise training and uninterrupted sleep (>6 hours). Pharmacological options include diuretics (thiazide-type diuretic and add-on loop diuretic if required for volume management), angiotensin neprilysin inhibitors, and β-blockers (carvedilol is preferred given its mortality benefits in patients with HF with additional BP lowering effects). Further steps include the use of spironolactone (MRA) (preferred due to PATHWAY-2 data), SGLT2i (for CVD risk reduction in HFrEF even without diabetes mellitus), and hydralazine + nitrate + CCBs (second-generation (amlodipine)) (non-dihydropyridine to be avoided). The use of α-blockers and minoxidil should be avoided. The focus of treatment should be to maintain a target heart rate of 50-60 bpm [44,45].

Importance of treatment compliance

The pill burden in RH patients is high as they are on >3 drug regimens, which often leads to non-compliance. The expert consensus states that a patient should comply with 80% of his prescribed dosage regimen. Around 7%-60% of RH patients are non-compliant with the treatment plan. Low rates (8%) of non-compliance are reported in studies monitoring prescription refills from the pharmacy, while serum drug monitoring for non-compliance has reported higher rates of 70%. Treatment compliance in every apparent treatment-resistant hypertension (aTRH) patient must be assessed (indirect (pill counts and self-report medication adherence assessment) and direct (serum drug monitoring)) and corrected if required. It will also help avoid unnecessary intensification of the treatment regime. Patient education and tools to monitor BP and treatment compliance will improve outcomes. Reasons for non-compliance with treatment in aTRH patients can vary widely, but some common factors include forgetfulness, side effects, cost of medication, lack of understanding about the importance of treatment, and difficulty incorporating treatment into daily routines. Addressing non-compliance requires a multifaceted approach, which may include educating patients about the importance of treatment and potential side effects, simplifying medication regimens, providing financial assistance or alternatives for expensive medications, offering reminder systems (such as alarms or pill organizers), involving family members or caregivers in the treatment plan, and regularly assessing and addressing any barriers to adherence through open communication and support. Additionally, healthcare providers can explore alternative treatment options or strategies tailored to individual patient needs and preferences to improve long-term adherence and treatment outcomes [46].

Patient engagement and education

Clinicians need to have sessions focused on engaging the patient, their family members, and caregivers in enhancing their knowledge about the disease, treatment, and factors influencing outcomes. Patient education should be done in both written and oral communication. It should be interactive with the use of visual materials. Methods such as "teach back" and motivational, encouraging, and blame-free environments can be used as appropriate. An alternative approach to patient education, particularly in situations with limited resources and time constraints, could involve leveraging technology. Utilizing interactive educational software or mobile applications can provide patients with access to comprehensive information at their convenience. These platforms can incorporate visual materials, interactive modules, and even virtual simulations to enhance understanding and engagement. Additionally, incorporating regular check-ins via telemedicine or messaging applications allows for ongoing support and clarification, fostering a sense of empowerment and accountability in patients' self-management of their health.

Renal denervation

Renal nerve ablation has been explored as a treatment approach over the past decade as there is a role for high sympathetic tone in RH. Despite promising results from small uncontrolled studies, the first sham-controlled randomized clinical trial, SYMPLICITY HTN-3, failed to show any benefit of renal nerve ablation. Several limitations of this trial led to widespread criticism. Since then, two studies, the Renal Denervation for Hypertension (DENERHTN) trial and the SPYRAL HTN-OFF MED (SPYRAL Pivotal) trial, have shown significant results in favor of renal denervation. However, further data is being generated before it becomes a standard of care in RH patients [47].

Future directions and newer agents

Management of RH has several unmet needs. These can be taken care of by generating new data and identifying more unique treatment options. Drugs such as angiotensin receptor-neprilysin inhibitors (ARNi) and non-steroidal MRA (finerenone) (fewer side effects) will help better manage RH and improve clinical outcomes. New agents including aldosterone synthase inhibitors (devoid of glucocorticoid activity), endothelin antagonists (combined endothelin A and B receptor blocker), and aminopeptidase A inhibitor that has central effects on vasopressin will likely provide additional treatment options on the completion of their clinical development [48]. The positive impact of flozins in reducing BP and improving clinical outcomes need to be confirmed in prospective trials.

## Conclusions

The burden of RH is on the rise globally as these patients are at a higher risk of major cardiovascular events and all-cause mortality than controlled HTN. With low rates of awareness and high dropouts at diagnosis, treatment, and control stages in India, the hypertensive patient is already at a significant behavioral disadvantage once diagnosed with RH. Managing RH requires a high degree of harmony between patients, physicians, and healthcare providers, resulting in an appropriate intensification of treatment, better compliance, and regular monitoring of BP. Patient engagement, education, and access to healthcare and medications will be the essential tools to manage HTN better and prevent its progression to RH.
